# Utilizing physical educators to monitor muscular strength and neuromuscular control among children with varied recess time

**DOI:** 10.3389/fspor.2025.1527810

**Published:** 2025-02-06

**Authors:** G. Kate Webb, Yan Zhang, Deborah J. Rhea

**Affiliations:** ^1^ Department of Kinesiology, Texas Christian University, Fort Worth, TX, United States; ^2^ Applied Health Science, Texas Christian University, Fort Worth, TX, United States

**Keywords:** physical education, children, muscular strength, neuromuscular control, recess, Hispanic, school

## Abstract

**Introduction:**

Inactivity levels among children are climbing at alarming rates, leading to a lack of physical activities that produce muscular strength (MusS) development, which in turn creates effective neuromuscular control (NC) development. Developing appropriate MusS during childhood decreases the chances of physical injuries and many chronic diseases such as type II diabetes and cancer, which leads to healthier, active future adults. The purpose of this study was to utilize the physical education setting to examine MusS and NC factors in the Fall and Spring (Time 1 to Time 2) of one school year in a predominately Hispanic sample of second-grade children who received 60 min or 20 min of daily recess.

**Methods:**

This quasi-experimental pre-test/post-test study administered four MusS tests and one NC test to District 1 (*N* = 59) which received 60 min of recess daily (intervention), and District 2 (*N* = 49) which received one 20 min daily recess (control). ANCOVAs were run for group differences at Time 2 while controlling for Time 1.

**Results:**

Intervention children significantly outperformed control children on the single leg 3-hop muscular strength test F(1,105) = 13.1, *p* < .001, *n*^2^ = .05, and the neuromuscular control side-step test F(1,105) = 4.77, *p* = .03, *n*^2^ = .04. Between group ANCOVAs controlling for body fat percentages showed the single leg 3-hop test remained significant between groups F(1,91) = 23.5, *p* < .001, *n*^2^ = .09.

**Discussion:**

Increased recess was shown to aid in improved lower body muscular strength and neuromuscular control among second grade children, even when controlling for body fat percentages. As 70% of American children are not participating in the recommended daily activity guidelines, and roughly 20% of American children are overweight, exploring movement opportunities for children and efficient means of monitoring MusS and NC is pivotal to future health and movement functions of children.

## 1 Introduction

Physical activity (PA) levels during childhood have declined as over 70% of American children do not participate in the Center for Disease Control and Prevention (CDC) recommended 60 min of PA, and roughly 20% are considered obese (1). Childhood physical inactivity hinders proper musculoskeletal strength as bones and muscles weaken through decreased limb use (2–5). Inactivity is highly problematic as muscular strength (MusS) is a dominant factor in producing neuromuscular control (NC), which is the brain's ability to recruit muscles efficiently for increased motor and movement skills and decreased injury (6–9). MusS is additionally essential for children due to its ability to help prevent diabetes, obesity, cardiovascular disease, bone mineral density, blood lipid profiles, insulin sensitivity, cancer, and mental health (10–14).

**Figure 1:**
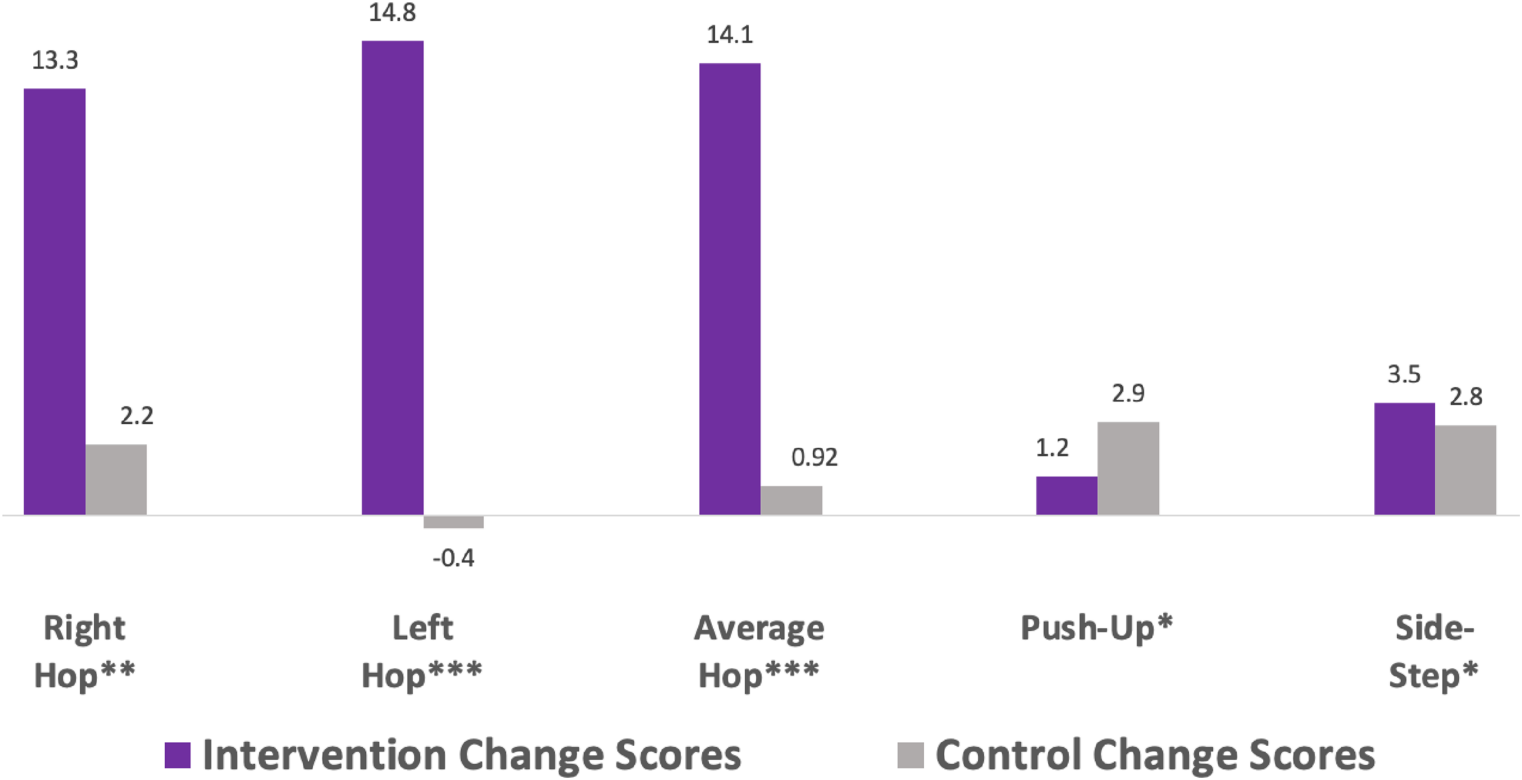
Change scores from time 1 to time 2.

Despite CDC guidelines, childhood inactivity is still prevalent, and intensified as PA inequalities exist across cultural backgrounds, sex, and body fat composition, presenting significant developmental gaps as children mature (15–23). For many children, varying cultural backgrounds and social health determinants play a key role in PA engagement (24). These PA disparities exist due to socio-economic and cultural influences, where those who are more advantaged tend to be more physically active, less sedentary, and therefore, less likely to suffer adverse health conditions (15, 23). Unhealthy housing, unsafe neighborhoods, and fewer convenient community PA locations reduce PA opportunities for varied child populations (25–27). Children in lower-income households appear to have less opportunity for PA participation, creating more significant developmental gaps in fine and gross motor, cognitive, language, and socio-emotional skills as they mature (18). The American Heart Association's “2022 Life Essential 8” assessed the cardiovascular health (CVH) of 74,435 American children ages 2–19 revealed white children (77.2%) participated in more PA and muscle-strengthening activities and were less overweight/obese than Black (75%) and Hispanic (71.1%) children. The study also revealed 78% of the boys, on average, participated in healthy PA while 71.8% of the girls, on average, participated in healthy PA, and girls had a 1.6% higher average BMI than boys (26). Sex plays an important role in a child's PA involvement as boys, on average, participate in roughly 40% more moderate to vigorous PA than females (28). This is a serious disadvantage to females, as MVPA is highly influential in maintaining healthy physical development markers during childhood (29, 30). These statistics leave Hispanic females in a category for the least movement opportunities, and therefore the least physically active demographic group.

PA disparities such as these lead to an increased childhood obesity risk (16, 20, 31). This weight gain accumulation creates an overall PA decline compared to normal-weight non-obese counterparts (7, 32–35). Obesity not only stresses children's joints, it also alters gait mechanics and increased hip pain, further limiting PA levels (19). Decreased PA levels have left American children with less bone mass and consequently less MusS than children four decades ago, preventing a means for lifetime activity (1, 36). Without the MusS foundation, NC is greatly hindered as well, and the ability to advance in movement skills is stifled (6, 8). This is not a hopeful diagnosis for millions of children entering what should be considered the most active time of their lives.

The conditions in which children are born, live, and play impact their PA opportunities, but school settings can provide safe, equitable access, and developmentally appropriate PA programs and movement opportunities daily for many children (7, 15). There are different ways to incorporate movement in the school day, but school recess, or unstructured, outdoor play, or recess, has unique child development benefits that may transcend PA inequalities (37). Recess within the school day provides movement opportunities for children regardless of demographics, income, or local extracurricular opportunities offered. Recess provides the freedom of choice in movements participated in, which is essential for PA opportunities among children of varied demographics, sex or body fat percentages. Although recess provides opportunity for increased PA, not all stakeholders in education understand the value of this entity. Therefore, not all children have access to safe recess facilities, nor have policies in place to assure children are receiving adequate recess time.

This is very unfortunate, as recess provides children with a perfect space to be physically active, coordinate limb movements, and move kinesthetically, while engaging in single or combined motor tasks like walking, running, jumping, dodging, and climbing movements (38–40). This freedom to move leads to increased MusS development naturally through play, which in turn, provides the foundation for motor coordination of gross and fine motor skills (5, 39, 40). These movements during play provide the confidence to engage in more advanced movements over time (38, 40, 41). Research also shows increasing movement opportunities, such as recess during the school day, can improve musculoskeletal traits for up to four years after physical activities in school have ended (36, 42).Therefore, time spent in recess should be considered foundational for proper physical development and MusS during the critical developmental elementary years.

Although previous research has shown recess to be a viable means for limb movement utilization that could enhance muscular and neurological development (40), the long-term effects of recess on MusS development still needs to be established. One successful longitudinal recess intervention showing whole child development is the LiiNK Project® (Let's inspire innovation ‘N Kids). The LiiNK Project implements four 15 min recesses and one 15 min character lesson (Positive Action®^)^ daily in elementary and middle schools (43). Though PA is not the primary goal of this recess project, the LiiNK research team has found moderate to vigorous PA (MVPA) occurs significantly more, postural balance increases and motor competencies increase as children participate in more unstructured, outdoor recess (38, 44, 45). Although many whole child benefits have been found through LiiNK's longitudinal intervention, the MusS and NC recess benefits have yet to be studied.

Whether genetically or environmentally driven, demographics such as sex, race, and body fat play a role in PA behaviors, increasing or decreasing MusS and NC development during childhood. Access to quality movement opportunities is essential for MusS, NC, and the overall health of every child (32). Physical educators' ability to assess MusS and NC development within childhood, and across various interventions or physical education programs is equally essential. Therefore, the purpose of this study was to use the physical education setting to examine MusS and NC factors in the Fall and Spring (Time 1 to Time 2) of one school year in a predominately Hispanic sample of second-grade children who received 60 min (LiiNK intervention) or 20 min (control) of daily recess. It was hypothesized that children in the intervention group would demonstrate greater improvements in MusS and NC scores compared to children in the control group. A second hypothesis was the intervention's positive effects on MusS and NC would be consistent even when controlling for body fat percentages between groups. The independent variable for this study was the recess group and the dependent variables were the mean scores for each of the six tests administered (single hand grip, single leg 3-hop, push-up, vertical jump, side-step, and body fat analysis).

## 2 Materials and methods

### 2.1 Participants

This quasi-experimental pre-test/post-test study focused on second grade students from two Texas public school districts (one elementary school per district) with predominately Hispanic populations. As previous studies have shown demographics can influence PA levels in childhood, the school districts were chosen due to similar demographic populations (24–27). District 1, the LiiNK intervention children (*N* = 59) received 60 min (four 15 min segments) of recess daily, while District 2, the control children (*N* = 49) participated in one 20 min daily recess. The children's distribution of sex, race and body fat percentages (*N* = 108) are provided in Table 1 and Table 2. Demographic data for sex and race were gathered from the participating school principals. Children's race was predominantly Hispanic in both groups, intervention (72%) and control (61%). Other races (White, Black and Asian) were combined as the “non-Hispanic” group for descriptive presentation. As race research has shown to influence child PA participation levels, the two districts were chosen for time spent in daily recess and the predominantly Hispanic populations. Time 1 assessments were completed in October 2023 and Time 2 assessments were completed in February 2024. The children were tested during their regularly scheduled physical education classes and all physical education classes took place between 9 and 10:30am. The inclusion criteria for participating in this study were (a) parent consent and child assent, (b) no injury that inhibited participation in the physical education class, and (c) being present on the day of testing.

**Table 1:** Sex and race distribution comparison between intervention and control groups.

Demographic Groups	*N*	Intervention (*N* = 59)	Control (*N* = 49)	*X* ^2^	*p*
Sex					
Female	57	32 (54%)	25 (51%)	.11	.74
Male	51	27 (46%)	24 (49%)		
Race					
Hispanic	73	43 (72%)	30 (61%)	1.66	.20
Non-Hispanic	35	16 (28%)	19 (39%)		

**Table 2:** Body fat category comparisons between intervention and control group*s.*

	Time 1		Chi-square or *t*-test	Time 2		Chi-square or *t*-test	Change scores^a^		Chi-square or *t*-test
Body fat categories	Intervention	Control		Intervention	Control		Intervention	Control	
Healthy	34%	43%	C^2^ = .78, *p* = .38	39%	41%	C^2^ = .04, *p* = .85	+5%	−2%	C^2^ = 2.68, *p* = .10
Overfat	20%	22%	C^2^ = .05, *p* = .82	25%	22%	C^2^ = .11, *p* = .74	+5%	0%	C^2^ = 1.89, *p* = .17
Obese	27%	31%	C^2^ = .17, *p* = .68	27%	35%	C^2^ = .68, *p* = .41	0%	+4%	C^2^ = 2.29, *p* = .13
Body Fat%	26.8 (10.7)	25.5 (8.6)	t(93)=−.60, *p* = .55	27.5 (11.0)	24. (8.9)	t(93)=−1.28, *p* = .20	0.77 (3.7)	−0.73 (1.9)	t(93)=−2.29, *p* = .02

Change from Time 1 to Time 2 (Time 2 scores minus Time 1 scores).

Intervention *n* = 57, Control *n* = 37.

### 2.2 Sample

*A priori* power analysis was conducted using G*Power 3.1.9.2. To detect a between-group effect, the power analysis was performed with alpha and beta set (α = 0.05; β = 0.80) and with a small effect size (*f* = 025) (46). The power analysis computed 128 participants for adequate statistical power. The original sample size was 80 participants per group (160 total) accounting for attrition between Time 1 and Time 2. In order to be included in the analysis, each child had to complete all pre-tests and post-tests. Due to absences on the post-test days, some children were removed from further analysis, leaving the final sample size at 108. Table 1 shows sex and race descriptives for the study. A chi-square analysis determined no differences between groups by sex and race. This analysis confirmed the two groups were similar for hypotheses testing.

### 2.3 Measures

Four MusS, one NC, and one body fat test were administered to children in both groups at Time 1 (October) and Time 2 (February). Muscular strength testing during childhood can be done either unilaterally (one limb singularly) or bilaterally (both upper or lower limbs working simultaneously). Four MusS tests (single hand grip, push-up, single leg 3-hop and vertical) assessed the unilateral and bilateral strength of the upper and lower limbs. MusS in childhood can also be exhibited as increased NC, or motor units recruited in more abundance and with greater efficiency (9), therefore, one NC test (side-step) was also included. With the rise in childhood obesity, Bioelectric Impedance Analysis (BIA) was included to better understand the intervention effects with this population. All six assessments identified for this study were found to be affordable (materials purchased online and totaled $120), portable, easy to use in the field such as a physical education setting, and showed good reliability in previous studies (33, 40, 45, 47–53). Additionally, the PI trained the physical educators to help administer the tests and aid in data collection.

#### 2.3.1 The digital dynamometer single hand grip test

The Single Hand Grip test is a highly reliable instrument used to assess hand/arm unilateral upper-body MusS (33, 47). Grip strength is associated with the prediction of musculoskeletal fitness, upper body strength, triglyceride levels, cardiometabolic health, cardiovascular disease, type II diabetes, and bone health during childhood and into adulthood (10, 47, 54, 55). As a current and future indicator of childhood health, grip strength was included in the MusS testing of children.

The GRIPIX Digital Dynamometer grip strength instrument was used to administer this test. To start the assessment, the tool was powered on and set to the appropriate age group and sex. The children were asked to stand with both feet shoulder-width apart, and maintain an angle of 15 degrees so that the torso and the arm (to be measured) did not touch each other, held the handle of the dynamometer with the second joints of their fingers, and pulled the handle while keeping their arms from shaking. They were asked to grip the dynamometer with their choice of whichever hand to begin. Once the instrument was checked to be in the correct position, the child was asked to squeeze the device for five seconds. The child was then asked to alternate hands and repeat the method. The grip strength of both hands was measured, twice on each side, and the highest value was recorded in pounds to the first decimal place. The highest attempt for the right and the left grip was used for statistical analysis.

#### 2.3.2 The single-leg 3-hop test

The Single-Leg 3 Hop Test assesses unilateral lower-body MusS and power in pre-adolescent children (49, 56). It is an easy, field-expedient, inexpensive test to administer, and is often used to indicate sport and physical activity readiness, as low scores in the single hop test are associated with increased injury risk in the thigh and knee (57). Additionally, research shows a strong relationship between low limb symmetry (distance hopped between limbs difference) and a propensity to develop foot and ankle injuries (58).

To set up for the assessment, a four-foot line of tape was placed on the floor to designate the start line. A 20-foot perpendicular line of tape ran from the center of the start line out the full 20 feet. A 20-foot tape measure was then placed with the zero at the back of the start line and taped down on each end. Children were taken through the following steps: (1) stand behind the start line with toes on the edge but not over; (2) balance on one foot of choice until stable; (3) hop as far as possible three consecutive times on a single leg without losing balance and landing firmly each time; and (4) on the third hop, stabilize on the landing foot for at least 2 s so the distance could be recorded. Failure to stick the final hop ended in a voided test score. The distance was measured from the start line to the heel of the landing leg down the perpendicular line. The children performed this process for two attempts per leg (right/left/right/left). Each distance was recorded to the nearest foot and whole inch. The farthest attempt for each leg was used for the analysis. If the child only had one attempt due to a voided test score, that score was used.

#### 2.3.3 The push-up test

This 90-degree Push-Up Test, as introduced in the FINTESS GRAM for American elementary school students, assesses upper-body bilateral MusS (50, 53, 59). During the push-up, the muscles in the front and back of the arms (triceps brachia and biceps), chest (pectoralis major), and shoulders (deltoids) are recruited (59). The push-up test can aid in monitoring this upper-body mass recruitment on both sides of the body, known as arm and shoulder girdle strength (50).

To start the assessment, children assume a prone position with hands slightly wider than shoulders, legs straight, and toes tucked under. They raise into a plank position (shoulders, hips, and ankles are in a straight line as if a plank was placed on them). A soft foam ball is placed in a circular holder under the child's chest. The children participate in one practice push-up as they are cued to extend their arms while keeping their legs and back straight in a plank position, then to drop down so the chest touches the ball at the base of the push-up (90-degree angle of the elbows), and then return to the straight-arm plank position. After the practice push-up, the children were allowed to rest for 30 s and then return to the straight-arm plank position. They were cued to start when the 30 s timer began. The children completed as many push-ups as possible within 30 s. For attempted push-ups to count, the body must stay in a plank position, the push-up must start and end in the extended arm position, and the chest must touch the ball on each downward attempt. The children participated in this test only once, and the whole number of completed push-ups in 30 s was used for the analysis.

#### 2.3.4 The double leg vertical jump test

The Double Leg Vertical Jump Test assesses bilateral lower-body MusS and is one of the most common tests to assess bilateral (33, 48), explosive movements in elementary-aged children (48, 60). Assessing these movements is important to determine if the child is ready for the explosive contractions of the muscle-tendon attachments in the lower limbs, as these movements are utilized in many play and sport arenas as the child advances in movement skills (9).

To set up for the assessment, “0” of the tape measure was secured to the wall at the gym floor base, then was secured vertically up the wall. The children followed the following protocol: (1) begin in an upright position perpendicular to the wall at the tape measure; (2) extend one hand and arm up the tape measure with feet flat on the floor (hip and armpit touching the wall)—this is the “reach” which was recorded in nearest half inch first; (3) in their perpendicular position to the wall with hand still raised from the “reach”, lower their hips until the knees bent around a 90° position; (4) finally, jump vertically with both feet as high as possible, i.e., extend the hips and reach with the fingertips. The recording in nearest half inch was based on the furthest finger touch on the tape measure. Two attempts were recorded. The vertical jump score was calculated to the nearest half-inch from the highest recorded jump minus the “reach.”

#### 2.3.5 The side-step test

The Side-Step Test assesses neuromuscular control (NC) of the lower body (33). It calls for entire core and lower body recruitment to complete the task, allowing for a better understanding of the brain-body connection. American Academy of Pediatrics has noted MusS gains in pre-adolescent children are displayed as neuromuscular advances rather than muscle hypertrophy, as seen in pubertal children (9).

To set up for the assessment, parallel lines at 24” in length were taped 27” apart on the floor. Children were asked to stand with both feet inside the two taped lines. Once the timer started, they stepped with the right foot outside of the right line and returned it as quickly as possible, then they stepped with the left foot outside of the left line and returned it as quickly as possible. Side-steps must alternate between right and left feet and one foot must always be on the ground (no jumping). Only one attempt of total whole number taps was recorded for the analysis.

#### 2.3.6 The bioelectric impedance analysis (BIA)

Bioelectric Impedance Analysis (BIA) assesses body fat percentages, and categorizes the children as heathy, overweight, or obese based on sex and age normative data (51). BIA is able to determine an estimate of fat mass, fat free mass, water weight, and bone density that shows a moderate to strong association with DXA results (51). This assessment has been recommended as an alternative body fat measure to body mass index (BMI) in children and adolescent fitness manuals such as the FITNESSGRAM to collect data in a school setting (53). This shift is due to reducing human error, convenience of using it in large group settings, and body fat percentage accuracy across different populations (45, 51–53). As obese and overweight children are in jeopardy of decreased PA which can lead to MusS and NC deficiencies (1, 7, 32–36), BIA is included to better understand MusS and NC findings of obese and overweight children who participate in varying amounts of recess.

To set up BIA, the scale was put on the floor surface, turned on, and synced to the computer that would record the child's full analysis. Once the scale was ready and assent was provided, each child stood on the metal plates of the scale with their shoes and socks off when their names were called. After about 15–20 s, the scale flashed a green light signifying that the measurement was complete, and students returned to the class activity. The PI then saved the data to the computer, disinfected the scale with alcohol wipes, and called the next child to stand on the scale so the process could be repeated.

### 2.4 Procedures

Following University Institutional Review Board approval of this study (1801-65-1801), Superintendents, Principals, and physical educators at each participating school approved the data collection. An IRB approved consent form was sent home to each parent for signed approval. Once all parents submitted their signed forms to the children's homeroom teachers, the primary investigator (PI) collected all forms of parents who approved their children to participate in each of the six assessments described in the measures section. Although the parent consented, their child still needed to sign an assent form prior to participation in each activity. The physical educator worked with the PI to make sure each student assented or declined participation in writing. Opt out reminders were given at the beginning of each class and prior to beginning each activity.

Prior to beginning the assessment phase, the PI scheduled adherence training for three other researchers who would help collect the data. Once the PI felt the others could competently administer the five tests and the BIA, the PI assigned one of the five stations to each of the three researchers. Each of the stations was set up around the perimeter of the gym during the regularly scheduled physical education classes. One station consisted of two tests that took the least time (the Dynamometer Grip Test and the Vertical Jump Test). The single-leg three-hop test, the push-up test, the side-step test and BIA were separate stations. The PI trained the physical educators the morning of data collection to administer the MusS and NC tests alongside the data collection team. Each physical educator showed competence for the administration of the test before they were allowed to assist the researchers in collecting the MusS and NC tests. On the testing day, children wore the required tennis shoes and activity clothes for participation in the physical education class.

Children were informed about the purpose and technique of each test within one large group setting. They were then separated into eight groups and assigned one of the four stations. Once at each station, the researchers gave detailed instructions on how to perform their test and allowed the children to ask questions before beginning (61). Once all children at each station completed the test, each group rotated to the next station, while the researchers and physical educators remained at their assigned stations. Children could stop participation at any time. If a child stopped before all five tests were completed, their data was eliminated from the analyses.

### 2.5 Data security and analysis

The data management personnel from the children's schools shared their demographic data through a protective link with the PI as a result of a school district agreement with the PI's university. Each child was assigned an ID number to preserve confidentiality, so names could no longer be linked with demographic information. The child's ID numbers replaced their names to analyze MusS and NC testing, BIA data, and all demographic data. Data was collected, processed, and complied with general data regulation procedures.

All data was cleaned and coded in Microsoft Excel and then analyzed using IBM SPSS version 29. All data were examined for outliers and missing scores and children who did not complete all four MusS and the one NC test were removed from the data set. The children who attempted the push-up test, but could not complete at least one push-up, remained in the data set with a score of zero. A test administration error in push-ups occurred when one of the raters counted push-ups inaccurately in Time 1. The error was not detected until Time 2 data collection, making it impossible to fix in the appropriate time window. This error resulted in a deletion of 17 samples from the original intervention group, leaving the remaining 59 for analysis. No other outliers were detected.

Sex and race distributions were examined by group and chi-square was used to analyze any group differences. Descriptive statistics were determined for MusS scores, NC scores, and for body fat category percentages at Time 1, Time 2, for the average scores (Time 1 plus Time 2 divided by two), and the change scores (Time 2 minus Time 1) by group. For descriptives, independent sample *t*-tests were run to determine group differences for the MusS and NC tests at Time 1, Time 2, for average scores, and change scores. To answer Hypothesis 1, a MANOVA was run to determine group differences for the MusS and NC tests at Time 2 controlling for Time 1. To answer Hypothesis 2, a MANCOVA was run to determine group differences for the MusS and NC tests at Time 2 controlling for Time 1 with body fat mean scores as a covariate.

## 3 Results

### 3.1 Demographic information

Preliminary chi-square analyses determined if the body fat category percentage was an influencing factor between groups at Time 1, Time 2, and the change scores (Time 2 minus Time 1) (Table 2). An independent sample *t*-test determined body fat percentage group differences for Time 1, Time 2, and the change scores (Table 2). Body fat category percentages were not different between groups at Time 1, Time 2, or the scores. Body fat percentage, however, was found to be significantly different between groups for the change scores. Although body fat category groups did not significantly differ on the change scores, notable shifts existed:

• 5% of the intervention group moved into the healthy category from Time 1 to Time 2
• 2% of the control group moved out of the healthy category from Time 1 to Time 2
• 4% of the control group moved into the obese category from Time 1 to Time 2

### 3.2 Hypothesis testing

#### 3.2.1 Hypothesis 1a

Independent sample *t*-tests determined means (M), standard deviations (SD) and group differences for the MusS and NC tests with *post hoc* (Bonferroni correction) to reduce risk of Type 1 error (Table 3).

• Intervention children significantly outperformed control children for Time 1, Time 2, and average scores on the right single hand grip, left single hand grip, average of right and left single hand grip, push-up; and the NC side-step test.
• Intervention children significantly outperformed control children on change scores, meaning greater improvement over time, on the right single leg 3-hop, left single leg 3-hop, average of right and left single leg 3-hop, and the NC side-step test.
• Control children had higher change scores on the push-up test than intervention children, but the control children scored much lower in the Fall than the intervention children.

**Table 3:** Muss and NC test mean and standard deviations by group*.*

	Time 1		Time 2		Average Scores^a^		Change Scores^b^	
	Intervention pre-test	Control pre-test	Intervention pos*t*-test	Control pos*t*-test	Intervention average	Control average	Intervention change scores^a^	Control change scores^a^
MusS Tests								
Right Grip^c^	23.7 (6.4)**	20.0 (5.4)	25.3 (5.7)**	22.4 (3.4)	24.5 (5.6)***	21.2 (4.0)	1.6 (4.5)	2.4 (4.4)
Left Grip^c^	22.7 (6.2)***	19.1 (5.0)	23.7 (5.5)*	21.5 (3.4)	23.2 (5.4)**	20.3 (3.7)	1.0 (4.2)	2.4 (4.4)
Average^a^^,^^c^	23.2 (6.1)***	19.6 (5.1)	24.5 (5.4)**	22.0 (3.1)	23.87 (5.5)***	21.3 (3.6)	1.3 (4.0)	2.4 (4.0)
Right Hop^d^	69.7 (30.2)	74.1 (24.0)	83.0 (30.4)	76.3 (18.0)	76.3 (29.2)	75.2 (17.8)	13.3 (16.6)**	2.2 (23.2)
Left Hop^d^	66.2 (32.7)	73.4 (22.8)	81.0 (29.0)	73.0 (17.8)	73.6 (28.8)	73.2 (17.6)	14.8 (22.6)***	−0.4 (20.7)
Average Hop^a^^,^^d^	67.9 (28.7)	73.8 (22.9)	82.0 (28.9)	74.7 (16.6)	75.2 (28.2)	75.0 (16.8)	14.1 (15.5)***	0.92 (20.6)
Push-Up	7.1 (5.5)***	0.5 (0.8)	8.3 (6.4)***	3.4 (4.9)	7.7 (5.7)***	2.0 (2.6)	1.2 (3.4)	2.9 (4.7)*
Vertical Jump^c^	6.8 (2.0)	6.6 (1.9)	7.3 (1.9)	7.3 (1.7)	7.1 (1.8)	7.0 (1.5)	0.5 (1.6)	0.7 (2.1)
Side-Step	23.2 (4.6)*	21.3 (5.0)	26.7 (5.2)*	24.1 (6.2)	24.9 (4.3)	22.7 (4.7)	3.5 (4.6)*	2.8 (6.5)

^a^ Average (Average of right and left limb scores).

^b^ Change Scores (Time 2 minus Time 1).

^c^ Recorded in pounds to the nearest tenth.

^d^ Recorded in inches to the nearest half-inch.

Average Scores (Time 1 plus Time 2 divided by 2).

Significant differences between group averages, *p = .05, **p = .01, ***p ≤ .001.

Intervention *n* = 59, Control *n* = 49.

#### 3.2.2 Hypothesis 1b

**Figure 2:**
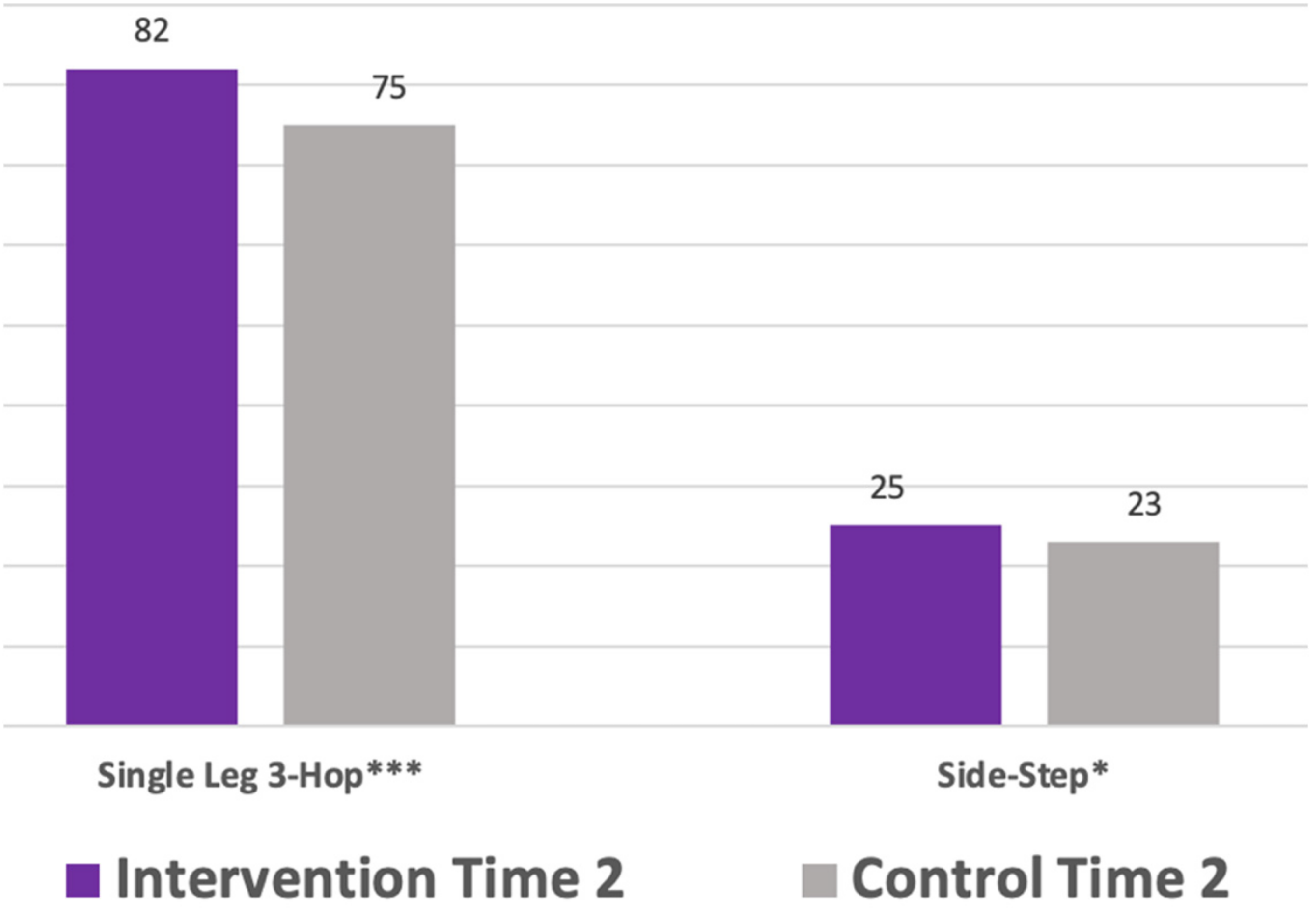
Significant differences in time 2 group scores controlling for time 1.

To establish Time 1 measures as an appropriate baseline between groups, Independent Sample *T*-Tests showed no significant group differences at Time 1 for the single leg 3-hop or the vertical jump. Significant group differences were found at Time 1 for the single hand grip, the push-up, and the side-step. Unable to use Time 1 as a baseline, for change scores, ANCOVAs determined group differences at Time 2 while controlling for Time 1 (Table 4, Figure 2).

• Time 2 scores, controlling for Time 1 showed intervention children significantly outperformed control children on the single leg 3-hop and the NC side-step test.

**Table 4:** Comparing time 2 measure of musS and NC by group, while controlling for time 1 & body fat percentages.

Main effect	MusS tests				NC Test
	Single hand grip	Single leg hop	Push-up	Vertical jump	Side-step
Group	F(1,105) = 2.68, *p* = .97, *n*^2^ = 1.7	F(1,105) = 13.1, *p* ≤ .001, *n*^2^ = .05	F(1,105) = 2.92*p* = .09, *n*^2^ = .01	F(1,105) = .26, *p* = .61, *n*^2^ = 00	F(1,105) = 4.77, *p* = .03, *n*^2^ = .04
Group controlling body fat	F(1,91) = 3.32, *p* = .07, *n*^2^ = .02	F(1.91) = 23.5, *p* < .001, *n*^2^ = .09	F(1,91) = 3.10, *p* = .08, *n*^2^ = .01	F(1,91) = .14 *p* = .71, *n*^2^ = .9.71	F(1,91) = 1.61, *p* = .21, *n*^2^ = .02

#### 3.2.3 Hypothesis 2

ANCOVAs determined group differences at Time 2, while controlling for Time 1, with body fat percentage as the covariate (Table 4) to answer Hypothesis 2.

• When controlling for body fat percentages, the NC side-step test was no longer significantly different between groups, but the MusS single leg 3-hop remained significant between groups.

## 4 Discussion

The purpose of this study was to use the physical education class setting to examine factors related to MusS and NC scores over time (Time, Time 2, average scores, and change scores) in a predominately Hispanic sample of second-grade children who received 60 min (LiiNK intervention) or 20 min (control) of daily recess. Research has shown Hispanic children, specifically Hispanic females, have less opportunity for PA and are more inactive than children of other races and sex (24–28). This study provides a unique contribution to literature with a focus on Hispanic children, movement opportunities accessible during the school day, and possible positive MusS and NC outcomes with the increased PA opportunities for this population of children.

Due to focusing on a predominantly Hispanic population across the two districts, race was not compared for differences. Conversely, sex and body fat were not found to be different at Time 1 or Time 2. As sex, race and body fat are shown in research to influence PA opportunities among children, the balanced sample allowed the intervention influence to be better observed (15, 16, 18–20, 23–25). Notably, we found consistent evidence for a positive association between increased time spent in daily recess with increased MusS and NC scores among this sample of predominantly Hispanic, second grade children.

LiiNK intervention children outperformed control children in Time 1, Time 2 and the average scores in single hand grip, the push-up and in the NC side-step (Figure 1). Although movement competency in early elementary years is essential for future movement skills, many studies report low levels of these skills among children (2–5, 62, 63). Second-grade children are a critical developmental age, as by age 7 they are expected to have engaged in adequate levels of movement competency such as running, jumping, kicking and throwing (64). Without this foundation, the ability to advance into more specialized skills such as sport or physical education is greatly hindered (6–9). As most children in this age group lack movement skills, the LiiNK intervention children were found to be more advanced in MusS and NC than their 20 min daily recess counterparts.

Controlling for Time 1, Time 2 scores were significantly better for the LiiNK intervention children in the single leg 3-hop (Figure 2). This significant improvement may indicate the extra time spent in recess readies both individual lower limbs for sport and PA while decreasing injury risk to the entire lower body chain (thigh, knee, ankle and foot). All MusS tests are important measures for assessing childhood fitness and MusS function, but the single leg 3-hop is unique in its assessment capabilities. The single leg 3-hop test is used to measure MusS and power in the individual lower limbs of pre-adolescent children; indicating sport and physical activity readiness, as low scores in the single hop test are associated with increased injury risk in the thigh and knee, ankle and foot (49, 56–58). As second grade children are in a pivotal motor development stage, the performance in the single leg 3-hop gives the LiiNK intervention children an advantage in lower-body motor development.

Controlling for Time 1, Time 2 scores were significantly better for the LiiNK intervention children in the side-step test measuring lower body NC (Figure 2). This means that LiiNK children, who are allowed 60 min of daily recess from early childhood on, may be more prepared cognitively and neurologically for advanced movements and sport skills by second grade. NC is the brain's ability to recruit muscles efficiently for increased motor and movement skills and decreased injury (6–9). Inactivity among children is problematic to NC, as development advances with limb movements (65). This finding once again highlights the importance of these brain-body connections needed by age 7 and the possible advantage to those who participate in more outdoor, unstructured free play.

Control children showed significantly better change scores in the push-up, yet they started and ended with a substantially lower percentage of children capable of completing at least one or more push-ups. By Time 2, only 53% of control children could complete at least one push-up, whereas 85% of the LiiNK children could complete at least one push-up. The push-up test was unique compared to the other four tests in that many control children scored a zero. This was not found in the other measures. The push-up test, as it recruits the core, arms, chest and back, is used in younger children as a discriminatory predictor of the potential participation in future athletic or advanced movement endeavors (66). Additionally, injury rates of children who participate in sport from ages 7–12 have increased in recent years, and is higher than adolescent and adult players (67, 68). This increase in child injury rates may be due to low skills and lack of MusS (69), once again highlighting the importance of MusS testing within this population. As upper body MusS is linked to decreased injury and increased sport readiness, it appears LiiNK children are more prepared for advanced upper-body movement with decreased chance of injury.

LiiNK intervention children shifted into healthier categories by 5% from Time 1 to Time 2 (overweight to healthy and obese to overweight), but had no shifts towards obesity. Control children shifted 2% from the healthy group to overweight or obese, had no change among the overweight group, and shifted 4% into the obese group from Time 1 to Time 2. Recess aids in increasing limb movements and MVPA for elementary-aged children (44, 61). Through recess, these PA increases may be more associated with lower body fat percentages in children (70, 71). Extra time spent in recess may be a factor in children shifting to the healthy body fat category and preventing a shift into the obese category over time.

When controlling for bodyfat, LiiNK intervention children continued to outperform control children in the single leg 3-hop. Increases of daily recess may allow children regardless of body fat percentages, to recruit and utilize lower body MusS to a significantly greater degree. Obese children are typically found to have less MusS, which hinders advanced movement skills, increases injury and decreases risk of all-cause mortality (7, 9–14, 19, 32–34). When controlling for body fat, children who participate in 60 min of daily recess maintained higher lower-limb MusS. This finding points to recess defined as unstructured, outdoor play as a potential PA opportunity to increase limb usage in children with higher body fat compositions especially in Hispanic populations.

## 5 Conclusions

PA discrepancies exist across race, sex and body fat percentages in children. A lack of equitable PA opportunities, where children of varied race, sex, and body fat compositions can participate is evident with the inactivity epidemic in America. Unstructured, outdoor play, or school recess, can provide an equitable PA opportunity due to its accessibility across demographics and its self-directed nature that spans comfort levels, cultures and intrinsic values of each individual child (72–74). The findings showed school recess to be a strong avenue for PA that has shown a preliminary positive influence on MusS and NC development among Hispanic, second grade children. This is an exceptional addition to the literature, as second grade students are at a pivotal developmental time in movement capabilities, where increases in MusS and NC set them up for more advanced sport and movement opportunities in the future, with less chance of injury. Whereas without this PA opportunity, typical trends within this age group point to movement deficiencies, leading to less MusS and increased injury. This study shows recess as a viable, equitable, PA opportunity across sex, race and obese children for MusS and NC advancement.

Feedback from the physical educators showed the MusS and NC tests were within the physical education class scope, and were simple to administer to students. For a class size of 50, the physical educator and one aid believed it would take no longer than one school week of regular scheduled physical classes (in Texas that is mandated at 150 min per week) to administer all tests. Students viewed the MusS and NC tests as fun “challenges” and repeatedly asked the PI and physical educators to repeat attempts for “fun” after the tests were completed.

Great MusS and NC gains were noted in this study among elementary children who received 60 min of daily recess, opposed to those who participated in 20 min of daily recess. To reap the benefits of whole child development, including appropriate MusS and NC development among a sedentary generation of children, it is recommended that elementary schools follow the *United Nations Convention on the Rights of the Child* (UNCRC) as a bare minimum recess policy (75):

• No less than 40 daily recess minutes separated into a minimum of two recess breaks daily
• Disallow withholding recess for missed school work and discipline
• Provide training for teachers and staff to ensure safe, healthy, outdoor, and inclusive recess

## 6 Future directions and limitations

In order for the results of this study to be generalized into larger population groups, this study should be repeated with a much larger sample size within a physical education setting across various demographics and geographical areas. In these larger studies, it would also be beneficial to collect at a minimum three time points (as opposed to two), as well as continuing to assess the intervention longitudionally. Additionally, this study was intended for typically developing children in elementary schools. The studies effects on non-typically developing children (those who need adaptations or inclusion teachers to aid in physical education activities) would be beneficial.

Although supplies for the MusS and NC tests are roughly $120 total, and therefore assessable in terms of cost, the testing protocol may not be easily understood by all physical educators. Therefore, the development of a training manual with video descriptions of how to administer procedures will need to be created if the tests are going to be assessable to all physical educators.

Other limitations were found in testing administration. The PI, three trained assistants, and trained physical educators administered the test, nevertheless, an error occurred in the push-up test that caused the deletion of 17 samples from the intervention group. This error occurred because the physical educator counted the push-ups similar to class procedures instead of the research training procedures. In order to prevent this in future studies, the PI and physical education teacher need to communicate clearly what a FITNESSGRAM push up looks like for research purposes, opposed to classroom protocol.

The last limitation was found in the vertical jump training. The PI noticed discrepancies in the ability to administer this test properly during training. Therefore, the PI solely administered the vertical test to control the potential for error. The PI suggests the two-foot vertical jump be replaced with the two-foot broad jump for future studies.

## Data Availability

The raw data supporting the conclusions of this article will be made available by the authors, without undue reservation.
